# Impact of Idiopathic Scoliosis on the Cardiopulmonary Capacity of Adolescents

**DOI:** 10.3390/jcm13154414

**Published:** 2024-07-28

**Authors:** Andrzej Siwiec, Małgorzata Domagalska-Szopa, Ilona Kwiecień-Czerwieniec, Agata Dobrowolska, Andrzej Szopa

**Affiliations:** 1 Child and Family Health Center in Sosnowiec, 41-218 Sosnowiec, Poland; 2 Department of Developmental Age Physiotherapy, Medical University of Silesia in Katowice, 40-752 Katowice, Poland; 3 Department of Physiotherapy, Medical University of Silesia in Katowice, 40-752 Katowice, Poland; 4 Neuromed, Rehabilitation and Medical Center, 40-698 Katowice, Poland

**Keywords:** cardiorespiratory fitness, exercise text, spinal deformity, VO_2max_

## Abstract

Chest deformities in children with scoliosis may result in cardiopulmonary disorders, lowering cardiopulmonary capacity levels. However, results regarding the dependence of cardiopulmonary capacity on the severity level of scoliosis remain controversial. We aimed to use dynamic cardiopulmonary exercise testing (CPET) to investigate exercise capacity in reference to spinal deformity in patients with mild or moderate idiopathic scoliosis by means of multivariate analyses. **Methods**: We included 92 children and adolescents aged 10–17 years with mild and moderate idiopathic scoliosis and 94 healthy peers. The study consisted of three parts: (1) medical record analyses; (2) anthropometric measurements; and (3) CPET. **Results**: Our results revealed that the mean VO_2peak_ and VO_2peak_/kg values of the participants with scoliosis were 0.27 L/min and 0.37 mL/min/kg lower than their healthy peers, respectively, regardless of age and gender. Furthermore, the occurrence of scoliosis correlates with a mean decrease in minute ventilation volume by 10.10 L/min, tidal volume by 0.11 L, breathing frequency by 3.78 bpm, and breathing reserve by 14.34%, regardless of the age and gender of the participants. Children and adolescents with mild-to-moderate scoliosis during CPET exhibit a lower ventilation capacity and lower VO_2_ max than healthy adolescents matched in age but their cardiorespiratory fitness parameters do not depend on the Cobb angle value and the angle rotation of the primary spinal curvature. **Conclusions**: Physical therapy and activity should be recommended to prevent cardiorespiratory failure in later life in patients with scoliosis. This study may also provide CPET reference values for healthy children and adolescents with scoliosis.

## 1. Introduction

Idiopathic scoliosis (IS) is a three-dimensional structural deformity of the spine that affects 1–3% of children, usually aged 10–16 [1]. Apart from the dominant lateral bending of the spine, the multidimensionality and multisegmentation of IS changes are also emphasized in the course of scoliosis. The symptoms of IS also include disorders of the anterior–posterior spinal curvatures in the sagittal plane and disorders of the vertebral rotations and torsions in the transverse plane, which directly affect the position and shape of other parts of the locomotor system, such as the chest [1].

Strict vertebral–costal joints in the thoracic region of the spine enhance mobility restrictions. Along with a change in the position and structure of the vertebrae, displacement, slope, and torsion of the chest may occur concurrently, resulting in the formation of a costal hump on the convex side of the curvature and a cavity on the concave side [2].

Chest deformities that affect the position of the ribs on the concave and convex sides of the curvature may limit the proper function of the respiratory muscles and chest mobility. Along with the progression of scoliosis, a change in the internal shape of the chest may occur and the lung on the convex side of the curvature may become flattened and compressed, resulting in complex cardiopulmonary disorders and lower cardiopulmonary capacity in children.

Restrictive pulmonary dysfunction occurs frequently among patients with scoliosis and can be diagnosed using traditional pulmonary function tests, such as static pulmonary function testing (PFT) [3]. Furthermore, a positive correlation between lung function and scoliosis severity in children, youths, and adolescents has been shown [4,5,6,7]. However, the results of studies on cardiopulmonary function in patients with scoliosis are ambiguous and, in some instances, controversial. Studies have confirmed that, during the course of scoliosis, reduced cardiopulmonary capacity occurs only in its severe type; that is, in those variants in which, according to Cobb, the angle of the lateral bending exceeds 40° [8,9,10,11,12]. Other sources report that such disorders already occur in mild and moderate scoliosis when the angle of the lateral bending does not exceed 30° [13]. However, the occurrence of lower-level cardiopulmonary function has not been confirmed, not only in cases when the angle of lateral bending had reached a high value [14,15] but also in cases of moderate and mild scoliosis [16].

Although these studies appear to imply reduced exercise performance in patients with scoliosis, their results are uncertain. Results regarding the dependence of cardiopulmonary capacity on the severity of scoliosis are controversial. This may be because diverse research tools are used in studies evaluating the capacity of patients with scoliosis. Although the cardiopulmonary exercise test (CPET) is the most reliable and objective method used to test the function and operation of the cardiopulmonary system [8] and the direct measurement of O_2_ (VO_2max_), referred to as the “golden standard”, is a widely accepted cardiopulmonary efficiency index, only a few studies include it as an evaluation tool for the efficiency level in patients with scoliosis [8,9,11,14,15]. Some reports include conclusions based on the evaluation of cardiopulmonary capacity using the incremental shuttle walking test (ISWT) [17,18], the physical work capacity test (PWC170) [19], or the 6-min walking test [13,20].

Previous studies were limited to the participation of patients with mild scoliosis (<20° Cobb) [9,16] as well as those with severe scoliosis (>45° Cobb) (8,11,14), predicting the VO_2max_ level through the submaximal physical test [16,19] without defining a reference group and comparing the results with the standards for sex and age [10,11]. However, the biggest limitation was disregarding (omitting) the results of the interactive influence of the participants’ sex and age on their exercise capacity. Perhaps because of such limitations in studies on this matter, their results have been inconsistent.

We, therefore, aimed to use a dynamic CPET to investigate exercise capacity and its reference to spinal deformity in patients with mild or moderate IS using multivariate statistics. We hypothesized that children with scoliosis have a lower cardiopulmonary capacity than their healthy peers. Moreover, it has been assumed that the cardiorespiratory fitness (CRF) parameters of children with scoliosis depend on the Cobb angle and the angle rotation of the primary spinal curvature.

## 2. Materials and Methods

This is a cross-sectional study. The study design, protocol, and consent forms were developed in accordance with the Code of Ethics of the World Medical Association (Declaration of Helsinki). This study was approved by the Ethical Committee of the Medical University of Silesia (PCN/CBN/0052/KB1/116/I/22/23) on 28 February 2023. Before each child’s examination, a parent or guardian was informed about the course of the study and the potential risks. Furthermore, the parents or guardians provided written informed consent. Apart from the consent of their parents/legal representatives or guardians, adolescents aged 16 years who acted as minors were required to independently grant their own consent.

In consideration of a priori, before starting the study, the minimum sample size was calculated using the G*power 3.1.9.4 program (Heinrich-Heine-Universität, Düsseldorf, Germany). Assuming a moderate effect size (f = 0.15) and 95% statistical power of this test, α = 0.05 and three predictors, the minimum study sample was estimated at 90 participants.

### 2.1. Participants

This study included 100 adolescents with mild or moderate IS who were patients of local pediatric rehabilitation centers and participated in rehabilitation camps. The patients were diagnosed with scoliosis and the severity of IS in each case was determined using the Cobb angle measured on a radiograph by a radiologist who remained unaware of the project. The main inclusion criteria were as follows: (1) adolescents (male and female) aged 10–17 (early and middle stage of adolescence) with an X-ray radiograph taken in the frontal view; (2) Cobb angle on the X-ray radiograph within the defined value of 10–40° in the major curvature (mild and moderate scoliosis); type III and IV King–Moe classification; (3) consent from a doctor to perform the CPET; (4) and the participant needed to be able to follow the study instructions. A total of 100 sex- and age-matched healthy controls were included in the control group.

All participants had been examined by a pediatric cardiologist and those (1) who had been diagnosed with a cardiovascular or a pulmonary condition that might have limited their exercise capacity (based on a detailed medical history) or were taking medication that might have influenced their cardiorespiratory fitness (e.g., inhaled corticosteroids and beta-agonists); (2) who had been involved in out-of-school sports activities; and (3) whose body mass index (BMI) for age Z-score for a particular sex was <−2.0, or >2.0, according to the WHO growth reference, were excluded [21].

Four participants from the study group and two from the control group were excluded due to the OLAF BMI-for-age score (percentile charts for growth and nutritional status assessment in Polish children and adolescents from birth to 18 years of age) and four from each group were excluded because they did not reach their peak exercise effort within 15 min. The final analysis included data from 92 participants and 94 controls. The participant characteristics are listed in Table 1.

### 2.2. Test Protocols

The treatment of children with scoliosis consisted of participation (twice a year) in Special Inpatient Rehabilitation (SIR), during which patients spend three weeks at a specialized health center, where they undergo intensive physiotherapeutic scoliosis-specific exercises (PSSE) (two hours per day). Moreover, patients declared participation in PSSE (on average 2 times a week), which included various forms of outpatient physiotherapies, mainly the Functional Individual Therapy of Scoliosis approach (FITS) from Poland [22]. Additionally, part of the participants (*n* = 12;13%) declared irregularly using Night Time Rigid Bracing (NTRB), i.e., wearing a brace mainly in bed (8–12 h per day). No participants reported other forms of bracing.

The study was conducted at the John Paul II Pediatric Centre (Sosnowiec, Poland) and consisted of three parts: (1) medical record analysis; (2) anthropometric measurements; (3) and a CPET. Examinations were performed in a single experimental room by the same experienced examiner (physiotherapist) and took place between 8 am and 11 am.

Pursuant to the analysis of medical records and radiographs, the following indicators were determined: (1) Cobb angle and (2) primary and secondary curvatures. Children with scoliosis who met the inclusion criteria were selected.

### 2.3. Anthropometric Measurements

Weight and height were measured using a Tanita HR-001 (Tanita Corporation, Tokyo, Japan). BMI, calculated as weight divided by height squared (kg/m^2^), and a percentile of the BMI Z-score and the OLAF BMI-for-age and sex relevant for school-aged children and adolescents, were calculated [23].

### 2.4. CPET

Currently, the best practice to assess CRF in the pediatric population is the CPET using the Godfrey protocol, which is a progressive and incremental maximum test performed using a cycle ergometer [24,25]. During the test, VO_2max_ is determined and breath-by-breath gas analysis provides a comprehensive evaluation of exercise ventilation and circulation. The maximum (VO_2max_) or peak (VO_2peak_) value refers to the greatest amount of O_2_ consumed during physical activity. The value of this parameter is given in liters or milliliters of O_2_ consumed in 1 min per kilogram of body weight and is assessed through the progressive ergospirometry test to exhaustion [26]. The VO_2max_ provides significant information concerning the tolerance of certain systems (circulatory, respiratory, musculoskeletal, and nervous systems) to maximum physical effort.

CPET, using the progressive ramp cycle ergometer protocol, was conducted for each participant. The MetaLyzer^®^ 3B with Breath-by-Breath technology (Cortex Biophysik GmbH, Leipzig, Germany) and two sizes of bicycle ergometers (Corival Pediatric, Lode BV, Groningen, The Netherlands) were used for the purposes of the examination, depending on the age and height of the participants. Before the examination, the height of the cycle ergometer seat was individually adjusted for each participant, who was equipped with a heart rate monitor belt and face mask of appropriate size.

The workload ramp was individualized using the Godfrey cycle ergometer protocol [24] to achieve maximum exertion within 10–15 min. For each participant, the first stage of the test consisted of a 2-min rest and a 2-min warmup with a 0 W workload. After that, the workload was steadily increased until reaching planned exhaustion, depending on the height of the participant as follows: (1) for patients with height under 120 cm, an increase of 10 W per minute; (2) 120–150 cm, a 15 W per minute; and (3) >150 cm, a 20 W per minute [24]. This time range was selected based on recent recommendations and reports that stated that the peak value for VO_2_ is maximized between the 10th and 15th min in ramp protocols [3]. Each participant was notified of the necessity to maintain a constant pace while on the cycle ergometer, i.e., approximately 60–80 revolutions per minute [27].

The ergospirometry test was considered finished when (1) a participant, despite the increasing intensity of the exercise, was in the plateau phase of VO_2_ for 30 s and (2) the respiratory exchange (RER) factor was >1.1 and the heart rate exceeded 85% of the age-based maximum rate. Moreover, the test was stopped once a participant showed symptoms of subjective exhaustion and, despite being verbally encouraged to do so, was not able to maintain a constant pedaling pace of 60 rpm on the cycle ergometer or did not reach a peak exercise effort within 10–15 min. In such cases, the participants were invited to undergo reexamination later.

Where the plateau at VO_2_ max was reached and maintained for at least 30 s, the maximum value of VO_2max_ was recorded. In cases where the participant was unable to reach VO_2max_, VO_2peak_ was recorded. Table 2 presents the measured and analyzed parameters of the exercise test.

### 2.5. Statistical Analysis

The first step of data analysis was conducted using basic descriptive statistics, along with a normal distribution analysis using the Kolmogorov–Smirnov test. All analyses were performed using IBM SPSS Statistics v.27 (IBM Corp., Armonk, NY, USA) and JASP v.13.0 (University of Amsterdam, Amsterdam, The Netherlands) and the level of statistical significance was set at *p* < 0.05. Overall, the variable distribution analysis showed that the distribution of the results was not consistent with a normal distribution. The normality of distribution analysis also proved the existence of outliers, i.e., the extreme values, that is, those outlying from the normal distribution of three times the interquartile range, which were replaced by the median values. The detailed descriptive statistics are shown in Table 1.

Several analyses were then performed with the aim of establishing whether the study group differed from the control group in terms of the level of certain capacity parameters and identifying the dependence between the quantitative features of scoliosis and those parameters that define the capacity of the participants. The analysis consisted of the following: (1) the characteristics of the study and control groups (age, height, body weight, BMI, and Z-BMI) and the characteristics of the study group with information about scoliosis, that is, the Cobb angle value (for the primary and secondary curvature), the vertebral rotation value according to Raimondi and skeletal maturity based on the Risser sign; (2) statistical analyses to examine the homogeneity of the experimental and control groups in terms of age, sex, height, and body weight; and (3) a proposal of a hierarchical regression model in which the explained variables are certain capacity parameters, while the predictors include the age of the participants, their sex (women vs. men), and their group (study or control).

## 3. Results

Although the study and control groups were homogeneous in terms of the distribution of sex and age (Table 3), the preliminary analyses showed a significant interactive influence of age and sex on the exercise capacity of the participants. To obtain normalized CRF parameters that were independent of body size, age, and sex, a multiple regression analysis of the data was conducted.

A hierarchical regression model was used, in which the predictors included the group (study or control), sex (woman vs. man), and age of the participants. Hence, dummy coding was conducted with the following values: 1 = study group, 0 = control group, 1 = male, and 0 = female. Age was introduced into the model as a quantitative variable. The detailed factors concerning the tested regression models are listed in Table 4. The tested models were examined in terms of the assumptions of the regression model: the variables were not collinear, there were no significant outliers, and in most cases, the variables were homoscedastic.

While interpreting the results of the general CRF parameters such as the work rate and time of the CPET (the total test duration and time required to reach VO_2_), it was observed that the occurrence of scoliosis allows for a statistically significant explanation of the variability of both time parameters, i.e., the total test duration (T) and (β = 0.19) and time required to reach VO_2_ (VO_2TIME_) (β = 0.18). The regression models explaining the T and VO_2TIME_ variables were well adjusted to the following data: F_T_ (3.182) = 8.77, *p* < 0.001, and F _VO2TIME_ (3.182) = 8.50, *p* < 0.001, and explained the 11% variability of the T parameter and the 12% variability of the VO_2TIME_ parameter. Although these results were statistically significant, they were considered poor. Moreover, a comparative analysis of the remaining results showed that the significant CPET parameter that differed between participants in the groups was CPET time. In comparison with the control group, the participants with scoliosis in the study group, regardless of their age and gender, had a mean 3.34 min longer total test duration and needed 2.85 more time to reach VO_2peak_ The strongest predictor for the total test duration was age, which indicates that the total test duration as well as time required to reach VO_2_ increased with the age of the participants, regardless of the occurrence of scoliosis (0.67 min and 0.58 min with every year, respectively). Moreover, both parameters were dependent on the sex of the participants. The total duration of the CPET and the time required to reach VO_2_ was 2.71 min quicker in boys than in girls (Table 4).

In turn, while analyzing the results concerning the metabolic and gas exchange parameters, it was observed that a group factor allows a thorough explanation of the variability of the VO_2peak_ parameter. The proposed regression model was well adjusted to the following data: *F* (3.182) = 36.07, *p* < 0.001; this helped to justify the 36% variation in the dependent variable. The strongest predictor of the VO_2peak_ level was also the age of a participant (*β* = 0.55); the obtained group effect is essential, negative, and moderate (*β* = −0.26). Because the equation for the proposed regression model is VO_2peak_ = 0.62 + 0.10 * (age in years) − 0.44 * (gender-men) + 0.27 * (scoliosis group), the obtained result indicates that the participants with scoliosis showed a mean 0.27 l/min lower level of VO_2peak_ regardless of their age and gender (Table 4). Furthermore, in the case of participants aged 10–17, VO_2peak_ increased by a mean of 0.10 l/min with every year and, in the studied population, the boys’ VO_2peak_ level was lower by 0.44 L/min than in the girls (Table 4). Although similar tendencies were related to the VO_2peak_/kg parameter, the recorded group effect (with simultaneous control for age and sex) was poor. No statistically significant correlations were observed between the other gas exchange parameters and the occurrence of scoliosis.

While analyzing the results concerning the ventilatory parameters (ventilatory system responses), a multiple regression model was performed again, where the predictors included the age and sex of the participants and the occurrence of scoliosis. The proposed regression model was well adjusted to the ventilatory parameters: F_VE_ (20.02, *p* < 0.001); F_VT_ (50.68, *p* < 0.001); F_BR_ (3.182) = 6.32, *p* < 0.001; and F_BF_ (7.58, *p* < 0.001). These values helped to justify a model of the 10%, 24%, 18%, and 10% variations in the dependent variable, respectively. The conducted analysis proved that the occurrence of scoliosis in the participants allows for a significant and negative explanation of the ventilatory parameter variability (β_VE_ = −0.27; β_VT_ = −0.15; β_BF_ = −0.17; β_BF_ = −0.34, respectively) and the obtained results can be interpreted as moderate. The results indicate that the occurrence of scoliosis correlates with the mean decrease in ventilatory parameters regardless of the age and sex of the participants: VE by 10.10 L/min; VT by 0.11 L; BF by 3.78 bpm; and breathing reserve (BR) by 14.34%. The detailed parameters of the regression models are listed in Table 4.

While analyzing the results of the correlation between CRF parameters and the characteristics of scoliosis, statistically significant dependencies between the Cobb angle of the primary curvature and the metabolic or gas exchange parameters, including VO_2peak_, VO_2_/kg, VE/VEO_2_, and VE/CO_2_, were not recorded (Table 5).

It was observed that, among the other CRF parameters, there were essential, moderate, and negative correlations in terms of the Cobb angle and VE/CO_2_, with the total duration of the CPET, indicating that the greater the angle of the primary curvature, the smaller the ventilation equivalent for carbon dioxide and the shorter the test duration. The strongest correlations were observed between CRF and skeletal maturity based on the Risser sign. They referred to gas exchange parameters, including VO_2peak_ and VO_2_/kg, as well as mechanical ventilation parameters (VE, VT, and BF) (Table 5).

## 4. Discussion

We aimed to determine whether children and adolescents with mild and moderate IS with the main thoracic curve show lower exercise capacity. The hypotheses that children with mild and moderate scoliosis have a lower cardiorespiratory fitness level and that their CRF parameters are dependent on the severity of scoliosis, which was expressed as the Cobb angle value of the primary curvature and the vertebral rotation value according to Raimondi, were tested in comparison with the results of their healthy peers. A verification of the first hypothesis has revealed three main findings. First, children and adolescents aged 10–17 and suffering from mild and moderate scoliosis with the main thoracic curve show a lower level of the basic metabolic and gas exchange parameters than their healthy peers; regardless of their age and gender, the VO_2peak_ values of the participants diagnosed with scoliosis were 0.27 L/min lower and the VO_2peak_/KG values were 0.37 L/min lower. Second, the occurrence of scoliosis correlated with a mean decrease in ventilatory parameters: minute ventilation volume (VE) by 10.10 L/min and tidal volume (VT) by 0.11 L, as well as the breathing frequency (BR) by 3.78 bpm and BR of 14.34%, regardless of the age and sex of the participants. Lastly, in comparison with the controls, the time parameters of the CPET in the case of the participants diagnosed with scoliosis were significantly longer, regardless of their age and gender; their total test duration was 3.34 min longer and, respectively, they needed 2.85 more time to reach VO_2_.

In the context of previous studies, although the CPET is currently the most reliable and objective method to assess CRF, few studies have applied it to evaluate the efficiency level in patients with scoliosis [8,11,14,15,16]. Furthermore, some of these studies included children and adolescents with severe scoliosis; that is, cases where the Cobb angle value was >45° and where the occurrence of deficits in CRF might have been more reasonably explained by restrictive pulmonary dysfunction features [8,11,14]. Only the study by Barrios et al. was conducted in a group that was like ours: children and adolescents with mild-to-moderate scoliosis (10° < Cobb angle > 45°) and at a similar age, i.e., aged 11–16 years [9]. Although this study was conducted on a treadmill using the ramp protocol, the obtained results were like ours in terms of the averages of certain CRF parameters for participants diagnosed with scoliosis and their healthy peers [9]. The results, in line with ours, prove that children and adolescents with IS are less tolerant to exercise testing with lower VO_2max_ for standardized body weight. The convergence of these results supports the hypothesis that children and adolescents with mild-to-moderate IS have worse CRF than their counterparts.

Patients with idiopathic scoliosis may not show cardiorespiratory fitness discomfort while performing daily activities or during regular physical activities. These potential CRF impairments could be revealed by ergospirometric tests. Therefore, CPET should be routinely used for the monitoring of cardiopulmonary function in patients with IS, as well as to determine appropriate physical activity for them.

The strength of our results is that we obtained them using the most reliable and objective method to assess the function and operation of the cardiopulmonary system, the CPET [3,8,9,11,14,15]. The indicated differences concerning the CRF level in children and adolescents with scoliosis, in comparison with the results of their healthy peers, were supported by an analysis of the parameters based on the direct measurement of O_2_ (i.e., VO_2peak_ and VO_2peak_/kg), which are generally accepted as the “golden standard” for this kind of assessment. However, what is most important is that the results indicating lower cardiorespiratory fitness in children and adolescents with scoliosis were obtained by comparison with their healthy peers (the control group) and the statistical analysis consisted of, among others, multiple linear regression analysis taking into account potential confounders such as age and sex.

The added value of the present study was the development of CPET reference values for healthy children and adolescents from southern Poland aged 10–17 years (the control group consisted of 94 participants). Similar results were shown in population studies on CPET reference values for children and adolescents in north-western Croatia [28].

However, the obtained results do not support the second hypothesis that the CRF parameters are dependent on the Cobb and rotation angles of the primary spinal curvature. The only essential dependencies concerned the positive correlation between the metabolic and gas exchange parameters, including VO_2peak_ and VO_2peak_/kg, as well as VE/VEO_2_, VE/VECO_2_, and the Risser test results. Owing to the strict correlation between skeletal maturity level and the age of the participants, these dependencies seem obvious. In the study group of children and adolescents with mild-to-moderate IS, patients with more extensive curvatures (i.e., those within 10–45° of the Cobb angle) did not show increased limitations regarding CRF.

Our study has some limitations, which primarily include a lack of evaluation of the exercise activity levels of the participants. Even though the treatment of children with scoliosis mainly consisted of participation in two 3-week rehabilitation camps, the CPET was conducted directly before the camp and the participants in both groups presented the same exercise activity level, which involved participation in physical education classes. This factor may marginally disrupt our results. To minimize the influence of additional exercise activities, those involved in out-of-school sports activities were excluded from the study.

## 5. Conclusions

Children and adolescents with mild-to-moderate scoliosis during CPET exhibited a lower ventilation capacity and lower VO_2max_ than age-matched healthy adolescents. Furthermore, lower ventilation capacity and lower VO_2max_ may be responsible for reduced exercise tolerance in adolescents with mild-to-moderate IS and the CRF parameters of children with mild-to-moderate scoliosis are not dependent upon the Cobb or rotation angles of the primary spinal curvature. Physiotherapy and physical activity should be recommended to prevent cardiorespiratory failure in later life in patients with scoliosis. The physiotherapy program should include not only physiotherapeutic scoliosis-specific exercises but also exercises aimed at improving cardiorespiratory function

## Figures and Tables

**Table 1:** Characteristics of the study and control groups, showing between-group comparisons of demographic characteristics.

Parameters	Study Group (*n* = 92)	Control Group (*n* = 94)	*t*	*p*
Age, years, mean ± SD (range)	13.30 ± 2.46 (10–17)	13.26 ± 2.42 (10–17)	0.68	0.74
Height, cm, mean ± SD (range)	156 ± 15.55 (131–182)	159.05 ± 12.16 (135–185)	0.87	0.31
Weight, kg, mean ± SD (range)	45.61 ± 17.00 (32.7–95.0)	47.49 ± 15.12 (33.7–95)	0.62	0.77
Z BMI	0.7 ± 1.3 (−1.8–2.0)	1.2 ± 0.8 (−2.0–2.0)	−6.25	<0.001
Girls N (%)	63 (68.5%)	64 (68.0%)		
Boys N (%)	29 (31.5%)	30 (32.0%)		
Cobb (p), mean ± SD (range)	20.90 ± 11 (11–40)			
Cobb (s), mean ± SD (range)	15.83 ± 8.87 (3–33)			
King–Moe classification				
3	63 (68.5%)			
4	29 (31.5%)			

p, (primary); S, (secondar); SD, standard deviation; range, minimum–maximum; *t*, *t* test; *p*, *p*-value.

**Table 2:** Cardiorespiratory fitness parameters.

Parameter	Abbreviation	Definition
Peak workload [W]	Wpeak	Maximal work rate achieved
Total test duration [min]	T	Total duration of the cardiopulmonary exercise testing. Longer duration of the cardiopulmonary exercise testing is associated with higher levels of exercise tolerance/aerobic capacity
Time to reach VO_2_ max [min]	VO_2max_ Time	Time to reach VO_2_ max
Heartrate peak (beats/min)	HR	Maximal heart rate at peak exercise
Metabolic and gas exchange parameters		
Absolute peak oxygen uptake [L/min]	VO_2peak_	VO_2peak_ is the highest oxygen intake that was sustained for at least 30 s during cycling
Normative values of peak oxygen uptake [%]	VO_2_ NORM	Normative reference values of absolute peak oxygen uptake
Peak oxygen uptake per body mass [mL/min/kg]	VO_2peak_/kg	VO_2peak_/kg is a peak oxygen uptake per body weight calculated by dividing VO_2peak_ by total body mass for at least 30 s during cycling
Peak oxygen pulse (mL/beat^−1^)	Peak O_2_pulse	VO_2_ ÷ heart rate, highest recorded value averaged over 30 s during exercise
Respiratory exchange ratio	RER	A ratio of the volume of CO_2_ being produced by the body to the amount of O_2_ being consumed
Ventilation equivalent for oxygen [L/min]	VE/VO_2_	The ventilation volume required for the consumption of 1 L of oxygen
Ventilation equivalent for carbon dioxide VE/VO_2_	VE/VCO_2_	The ventilation volume required to remove 1 L of carbon dioxide
Ventilation parameters		
Minute ventilation volume [L/min]	VE	The ventilation in liters per minute
Tidal volume [L]	VT	The volume of gas inspired and expired during one respiratory cycle
Breathing frequency [bpm]	BF	The number of breaths per minute when working at a maximum intensity. Anaerobic threshold and peak exercise in breaths per minute
Breathing reserve [%]	BR	The difference between the maximal voluntary ventilation (MVV) and the maximum exercise ventilation (VE) in absolute terms, or this difference as a fraction of the MVV

**Table 3:** Cardiorespiratory fitness parameters characteristic of the study and control groups.

Parameters	Group					
	Control		Study		Control	Study
	Mean ± SD	Range	Mean ± SD	Range	S–W; *p*	S–W; *p*
Wpeak [W]	156.64 ± 93.03	91–198	178.23 ± 47.82	94–193	0.61; <0.001	0.72; <0.001
T [min]	10.05 ± 4.44	7.27–14.58	10.93 ± 4.06	9.51–15.00	0.64; <0.001	0.82; <0.001
VO2 TIME [min]	8.36 ± 5.99	8.24–14.57	8.53 ± 5.95	9.45–14.00	0.66;<0001	0.82; <0.001
HR_peak_ (beats/min)	181.68 ± 18.04	112–210	178.41 ± 15.58	113–206	0.89; <0.001	0.91; <0.001
VO_2peak_ [L/min];	1.51 ± 0.59	0.53–3.58	1.46 ± 0.43	0.74–3.40	0.87; <0.001	0.88; <0.001
VO_2_ %NOR	66.99 ± 17.25	50.61–76	62.06 ± 21.04	40.53–99.00	0.93; <0.001	0.86; <0.001
VO_2peak_/kg [mL/kg/min]	43.04 ± 8.34	21–52	33.59 ± 7.45	19–51	0.99; 0.671	0.97; 0.043
VO_2_/HR	8.28 ± 2.93	4–19	8.13 ± 2.26	5–19	0.85; <0.001	0.85; <0.001
RER	0.92 ± 0.08	0.83–1.10	0.97 ± 0.10	0.85–1.02	0.92; <0.001	0.83; <0.001
VE/VO_2_ [L/min]	30.28 ± 4.49	20.60–42.30	30.61 ± 5.26	21–43	0.98; 0.252	0.97; 0.071
(VE/VCO_2_) [L/min]	29.58 ± 3.50	21.80–35.10	29.20 ± 3.95	15.80–38.50	0.98; 0.157	0.97; 0.017
VE [L/min]	53.12 ± 21.01	15.30–132.10	51.32 ± 16.10	21.90–103.00	0.89; <0.001	0.96; 0.005
VT [mL]	1.14 ± 0.42	0.64–2.67	1.27 ± 0.33	0.67–2.60	0.87; <0.001	0.93; <0.001
BF [bpm]	37.27 ± 10.86	19–55	40.92 ± 10.38	23–69	0.99; 0.567	0.96; 0.007
BR%	43.63 ± 22.37	21–78	54.46 ± 17.74	50.50–82.00	0.86; <0.001	0.83; <0.001

M, mean; SD, standard deviation; range, minimum–maximum; S–W Shapiro–Wilk test; *p*, *p*-value. Wpeak, maximal work rate achieved; T, total test duration; VO_2Peak_, absolute peak oxygen uptake; VO_2_ NORM, normative values of peak oxygen uptake; VO_2_/kg, VO_2peak_/kg peak oxygen uptake per body mass; RER, maximal respiratory exchange ratio; VO_2max_, time to reach VO_2_ max; HR_peak_, maximal heart rate at peak exercise; BF, breathing frequency; BR%, breathing reserve; VE, minute ventilation volume; VT, tidal volume; VE/VO_2_, ventilation equivalent for oxygen; VE/VCO_2_, ventilation equivalent for carbon dioxide.

**Table 4:** Coefficients of multiple linear regression of the correlations between group, age, sex, and cardiorespiratory parameters.

Auth−Dependent Variables	Predictors	Unstandardized Coefficient		Standardized Coefficient	*t*−Value	*p*−Value	95% CI for B		R^2^
		B	Std. Error	β			LL	UL	
Wpeak	(Constant)	72.67	68.89		1.05	0.293	−63.25	208.59	0.23 **
	Age	17.63	3.39	0.40	5.20	<0.001	10.94	24.32	
	Sex	−63.64	17.32	−0.24	−3.67	<0.001	−97.81	−29.47	
	Group	−19.74	19.65	−0.08	−1.00	0.316	−58.52	19.04	
T	(Constant)	9.02	5.19		1.74	0.084	−1.23	19.26	0.11 **
	Age	0.67	0.26	0.21	2.62	0.010	0.17	1.17	
	Sex	−2.71	1.31	−0.15	−2.08	0.039	−5.29	−0.14	
	Group	3.34	1.48	0.19	2.25	0.025	6.26	0.42	
VO_2_ TIME	(Constant)	7.77	4.55		1.71	0.090	−1.22	16.75	0.12 **
	Age	0.58	0.22	0.21	2.59	0.010	0.14	1.02	
	Sex	−2.39	1.15	−0.15	−2.09	0.038	−4.65	−0.13	
	Group	2.85	1.30	0.18	2.20	0.029	5.41	0.29	
HR	(Constant)	181.90	10.38		17.52	<0.001	161.41	202.38	
	Age	−0.15	0.51	−0.03	−0.29	0.769	−1.16	0.86	
	Sex	−2.26	2.61	−0.07	−0.87	0.388	−7.41	2.89	
	Group	2.39	2.96	0.07	0.81	0.420	−3.45	8.24	
Metabolic and gas exchange parameters									
VO_2_ Peak	(Constant)	0.92	0.25		2.46	0.015	0.12	1.12	0.36 **
	Age	0.10	0.01	0.55	7.94	<0.001	0.07	0.12	
	Sex	−0.44	0.06	−0.41	−6.87	<0.001	−0.56	−0.31	
	Group	−0.27	0.07	−0.26	−3.75	<0.001	0.13	0.41	
VO_2_ NOR	(Constant)	39.04	11.76		3.32	0.001	15.85	62.24	0.02
	Age	0.99	0.58	0.15	1.71	0.089	−0.15	2.13	
	Sex	1.00	2.96	0.03	0.34	0.736	−4.83	6.83	
	Group	−8.13	3.35	−0.21	−2.42	0.016	1.51	14.75	
VO_2_ Peak/kg	(Constant)	42.11	4.73		8.91	<0.001	32.77	51.43	0.07 **
	Age	−0.48	0.23	−0.17	−2.07	0.040	−0.94	−0.02	
	Sex	−3.21	1.19	−0.20	−2.70	0.008	−5.55	−0.87	
	Group	−0.39	1.35	−0.03	−0.29	0.771	−2.27	3.05	
VO_2_ HR	(Constant)	3.96	1.24		3.18	0.002	1.51	6.41	0.40 **
	Age	0.52	0.06	0.57	8.50	<0.001	0.40	0.64	
	Sex	−2.35	0.31	−0.44	−7.52	<0.001	−2.97	−1.74	
	Group	1.29	0.36	0.25	3.65	<0.001	0.59	1.99	
RER	(Constant)	1.00	0.06		17.72	<0.001	0.89	1.12	0.06 *
	Age	0.006	0.003	0.18	2.15	0.033	<0.001	0.01	
	Sex	0.004	0.01	0.02	0.28	0.782	−0.02	0.03	
	Group	−0.02	0.02	−0.13	1.50	0.136	−0.06	0.01	
VE/VO_2_	(Constant)	21.32	2.91		7.34	<0.001	15.59	27.06	0.06 *
	Age	0.36	0.14	0.21	2.50	0.013	0.08	0.64	
	Sex	1.98	0.73	0.20	2.71	0.007	0.54	3.42	
	Group	1.13	0.83	0.12	1.36	0.174	−0.51	2.77	
VE/VCO_2_	(Constant)	22.84	2.20		10.36	<0.001	18.49	27.18	0.08 **
	Age	0.12	0.11	0.09	1.09	0.278	−0.10	0.33	
	Sex	2.13	0.55	0.28	3.85	<0.001	1.04	3.22	
	Group	1.13	0.63	0.15	1.80	0.073	−0.11	2.37	
Ventilatory system responses									
VE	(Constant)	23.94	10.04		1.39	0.167	−5.87	33.74	0.24 **
	Age	3.33	0.49	0.51	6.74	<0.001	2.36	4.30	
	Sex	9.92	2.52	0.26	3.93	<0.001	−14.90	−4.93	
	Group	−10.10	2.86	−0.27	3.53	<0.001	4.45	15.75	
VT	(Constant)	1.30	0.18		1.69	0.093	−0.05	0.64	0.45 **
	Age	0.09	0.01	0.70	10.90	<0.001	0.08	0.11	
	Sex	−0.23	0.04	−0.29	−5.12	<0.001	−0.31	−0.14	
	Group	−0.11	0.05	−0.15	2.26	0.025	0.02	0.21	
BF	(Constant)	49.40	6.44		7.67	<0.001	36.69	62.11	0.10 **
	Age	−0.77	0.32	−0.20	−2.44	0.016	−1.40	−0.15	
	Sex	−1.14	1.62	−0.50	−0.71	0.482	−4.34	2.05	
	Group	−3.78	1.84	−0.17	2.06	0.041	0.15	7.41	
BR	(Constant)	51.73	12.89		6.65	<0.001	57.48	105.97	0.18 **
	Age	−1.31	0.61	−0.18	−2.17	0.031	−2.51	−0.12	
	Sex	2.65	3.09	0.06	0.86	0.393	−3.45	8.74	
	Group	−14.34	3.51	−0.34	−4.09	<0.001	−21.25	−7.42	

B, beta; SE, standard error; β, differentiated beta; *t*, *t*-test coefficient; *p*, statistical significance of the *t*-test; LL, lower confidence interval; UL, upper confidence interval for beta; R^2^, specific R^2^ value; SI, symmetry index. *, ** Statistically significant values.

**Table 5:** Correlation between cardiorespiratory fitness (CRF) parameters and characteristics of scoliosis.

CRF Parameters	Cobb	Risser	Raimondi
	r Pearson; *p*-Value		
WR	−0.16; 0.121	0.09; 0.378	−0.11; 0.278
VO_2_ Peak	−0.03; 0.774	0.388 *; 0.047	−0.02; 0.828
VO_2_% NOR	0.17; 0.110	0.06; 0.571	−0.10; 0.335
VO_2_/kg	0.11; 0.276	0.373 *; <0.001	−0.14; 0.182
VO_2_/HR	0.02; 0.871	0.208 *; 0.047	−0.16; 0.120
VE	0.08; 0.471	0.220 *; 0.035	−0.07; 0.493
VT	−0.08; 0.462	0.451 *; <0.001	0.00; 0.971
BF	0.06; 0.555	−0.255 *; 0.014	−0.03; 0.767
BR%	−0.05; 0.643	−0.09; 0.374	−0.02; 0.865
VE/VEO_2_	0.16; 0.139	0.289*; 0.005	−0.09; 0.401
VE/CO_2_	0.19; 0.063	0.281*; 0.007	−0.13; 0.218
TIME	−0.314 *; 0.006	0.13; 0.208	−0.01; 0.961
VO_2_ TIME VE/CO_2_	−0.306 *; 0.030	0.13; 0.215	0.14; 0.182

* Statistically significant values.

## Data Availability

The datasets generated during and/or analyzed during the current study are not publicly available because the data are part of an ongoing study but are available from the corresponding author (aszopa@sum.edu.pl).
